# The potential role of RNA N6-methyladenosine in primary Sjögren’s syndrome

**DOI:** 10.3389/fmed.2022.959388

**Published:** 2022-11-16

**Authors:** Qiufeng Xiao, Xunyao Wu, Chuiwen Deng, Lidan Zhao, Linyi Peng, Jiaxin Zhou, Wen Zhang, Yan Zhao, Yunyun Fei

**Affiliations:** ^1^Department of Rheumatology and Clinical Immunology, Peking Union Medical College Hospital, Chinese Academy of Medical Sciences and Peking Union Medical College, Beijing, China; ^2^National Clinical Research Center for Dermatologic and Immunologic Diseases, Ministry of Science and Technology, Beijing, China; ^3^State Key Laboratory of Complex Severe and Rare Diseases, Peking Union Medical College Hospital, Chinese Academy of Medical Sciences and Peking Union Medical College, Beijing, China; ^4^Key Laboratory of Rheumatology and Clinical Immunology, Ministry of Education, Beijing, China; ^5^Clinical Biobank, Department of Medical Research Center, Peking Union Medical College Hospital, Chinese Academy of Medical Sciences and Peking Union Medical College, Beijing, China

**Keywords:** primary Sjögren’s syndrome, N6-methyladenosine, METTL3, ALKBH5, YTHDF readers, ISG15

## Abstract

**Objective:**

The pathogenesis of primary Sjögren’s syndrome (pSS) remains incompletely understood. The N6-methyladenosine (m6A) RNA modification, the most abundant internal transcript modification, has close associations with multiple diseases. This study aimed to investigate the role of m6A in patients with pSS.

**Materials and methods:**

This study enrolled 44 patients with pSS, 50 age- and gender-matched healthy controls (HCs), and 11 age- and gender-matched patients with non-SS sicca. We detected the messenger RNA (mRNA) levels of m6A elements (including METTL3, WTAP, RBM15, ALKBH5, FTO, YTHDF1, YTHDF2, YTHDF3, YTHDC1, and YTHDC2), ISG15, and USP18 in peripheral blood mononuclear cells (PBMCs) from patients with pSS, patients with non-SS sicca, and HCs. The clinical characteristics and laboratory findings of patients with pSS and patients with non-SS sicca were also collected. We used binary logistic regression to determine if m6A elements were risk factors for pSS.

**Results:**

The mRNA levels of m6A writers (METTL3 and RBM15), erasers (ALKBH5 and FTO), and readers (YTHDF1, YTHDF2, YTHDF3, YTHDC1, and YTHDC2) were all significantly higher in PBMCs from patients with pSS than in HCs. The mRNA levels of m6A writers (METTL3 and WTAP) and readers (YTHDF2, YTHDF3, and YTHDC2) were lower in PBMCs from patients with pSS compared to patients with non-SS sicca. The expression of METTL3, RBM15, FTO, YTHDF1, YTHDF2, YTHDC1, and YTHDC2 was positively correlated with the level of C-reactive protein (CRP) of patients with pSS. The mRNA level of YTHDF1 in PBMCs from patients with pSS was negatively correlated with the EULAR Sjögren’s syndrome disease activity index (ESSDAI) score. In patients with pSS, FTO, YTHDC1, and YTHDC2 were also related to white blood cells (WBCs), neutrophils, lymphocytes, and monocytes. Increased mRNA level of ALKBH5 in PBMCs was a risk factor for pSS, as determined by binary logistic regression analysis. The mRNA level of ISG15 was positively correlated with that of FTO, YTHDF2, YTHDF3, and YTHDC2 in patients with pSS.

**Conclusion:**

Compared with HCs, the expression of METTL3, RBM15, ALKBH5, FTO, YTHDF1, YTHDF2, YTHDF3, YTHDC1, and YTHDC2 was considerably higher in PBMCs from patients with pSS. In comparison with patients with non-SS sicca, the expression of METTL3, WTAP, YTHDF2, YTHDF3, and YTHDC2 was reduced in PBMCs from patients with pSS. The m6A elements correlating with clinical variables may indicate the disease activity and inflammation status of pSS. Elevated expression of ALKBH5 was a risk factor for pSS. The dynamic process of m6A modification is active in pSS. m6A elements (FTO, YTHDF2, YTHDF3, or YTHDC2) might target ISG15, stimulate the expression of ISG15, and activate the type I IFN signaling pathway, playing an active role in initiating the autoimmunity in pSS.

## Introduction

Primary Sjögren’s syndrome (pSS) is a chronic systemic autoimmune disease. It often occurs at the age of 50 years and has an apparent female predominance, with the male-to-female ratio of approximately 1:9 (1). Due to the lymphoid infiltration and progressive destruction of the salivary and lacrimal glands, patients with pSS usually suffer from oral and ocular dryness symptoms (2). Furthermore, pSS can have systemic manifestations, such as fatigue, articular symptoms, pulmonary disease, renal involvement, peripheral neuropathy, cutaneous lesion, and even B-cell lymphoma (3). pSS is characterized by its immunologic abnormalities, including the emergence of serum anti-SSA antibodies and focal lymphocytic sialadenitis (4). Therapeutic strategies for pSS include relieving the oral and ocular dryness symptoms and managing more severe organ involvement (5). pSS can significantly reduce health-related quality of life and can even lead to severe functional disability. However, the pathogenesis of pSS remains incompletely understood, and the treatments are still limited.

Over the past few years, epigenetic modifications have been found to regulate gene expression and are associated with various diseases. DNA methylation, histone modifications, and non-coding RNAs play an increasingly important role in the pathogenesis of pSS (6). However, RNA modifications have rarely been investigated in pSS. N6-methyladenosine (m6A) modification is the most common internal transcript modification in eukaryotic messenger RNA (mRNA) (7). The critical elements for m6A modifications mainly consist of m6A methyltransferase (writers), m6A demethylase (erasers), and m6A RNA-binding proteins (readers), which can install, remove, or recognize m6A on RNAs, respectively (8, 9). Writers are composed of METTL3, METTL14, WTAP, RBM15/15B, etc. The only two m6A erasers are FTO and ALKBH5. YTHDF1, YTHDF2, YTHDF3, YTHDC1, and YTHDC/2 are readers that contain the YTH domain (10). These proteins affect the process of m6A on target RNA and influence its transcription, localization, maturation, translation, degradation, metabolism, and function, thus modulating the expression of the target gene (11).

Numerous studies have shown that m6A modification has close associations with multiple diseases and especially plays an important role in tumor initiation and progression (10). Interestingly, recent studies have found that m6A may be involved in the pathogenesis of autoimmune diseases, including rheumatoid arthritis (RA) and systemic lupus erythematosus (SLE) (12–16). Wang et al. (12) discovered that the mRNA level of METTL3 was significantly elevated in patients with RA and had positive correlations with inflammatory markers, including erythrocyte sedimentation rate (ESR) and C-reactive protein (CRP). Besides, METTL3 promotes the inflammatory response of macrophages in patients with RA through the NF-κB signaling pathway (12). In addition, Luo et al. (15) concluded that decreased mRNA levels of ALKBH5, FTO, and YTHDF2 in peripheral blood mononuclear cells (PBMCs) are risk factors for RA. Moreover, two studies reported reduced mRNA levels of METTL3, MTEEL14, WTAP, ALKBH5, FTO, and YTHDF2 in PBMCs from patients with SLE. Furthermore, they revealed that reduced mRNA levels of ALKBH5 and YTHDF2 were risk factors for SLE (13, 14).

Nevertheless, the role of m6A in patients with pSS has not been studied yet. To determine whether m6A modification impacts the pathogenesis and development of pSS, we detected the mRNA levels of METTL3, WTAP, RBM15, FTO, ALKBH5, YTHDF1, YTHDF2, YTHDF3, YTHDC1, YTHDC2, ISG15, and USP18 in peripheral blood from patients with pSS, patients with non-SS sicca, and healthy controls (HCs). To ascertain whether m6A can reflect disease activity, we performed a correlation analysis between m6A and clinical indicators of patients with pSS and patients with non-SS sicca. We also explored the connections between m6A and interferon-stimulated genes (ISGs), which might provide a new perspective on how m6A influences the development of pSS.

## Materials and methods

### Patients and healthy controls

From April 2021 to October 2022, we enrolled 44 patients who satisfied the diagnosis of pSS according to the 2016 American College of Rheumatology/European League Against Rheumatism classification criteria for pSS (4). In addition, we enrolled 11 age- and gender-matched patients without SS sicca as a symptom control group. The recruitment of patients was conducted at the clinic of Peking Union Medical College Hospital. The exclusion criteria of this study included those patients who overlapped with other autoimmune diseases or suffered from other active inflammatory diseases or malicious diseases. Among the pSS group, 15 patients were treatment-naive, 23 patients were under hydroxychloroquine treatment, and 6 patients received prednisone monotherapy within 6 months. The clinical information of pSS was collected, including clinical symptoms, imaging examination findings, and laboratory results such as ESR, CRP, complement 3 (C3), complement 4 (C4), immunoglobulin G (IgG), immunoglobulin A (IgA), immunoglobulin M (IgM), rheumatoid factor, anti-SSA antibody, anti-SSB antibody, antinuclear antibody (ANA), WBC, red blood cell (RBC), hemoglobulin (HGB), platelet (PLT), lymphocyte, lymphocyte%, monocyte, monocyte%, neutrophil and neutrophil%. The disease activity of patients with pSS was assessed by the EULAR Sjögren’s syndrome disease activity index (ESSDAI) (17, 18). The HC group was comprised of 50 healthy individuals without autoimmune or inflammatory diseases. Age and gender were comparable between the pSS group, non-SS sicca group, and HC groups. The clinical data of the enrolled participants are listed in Table 1 and Supplementary Table 1. This study has obtained the approval of the Ethics Committee of the Peking Union Medical College Hospital and was carried out in compliance with the Declaration of Helsinki. Every participant provided informed consent before initiating the study.

**TABLE 1:** Clinical details of patients with primary Sjögren’s syndrome (pSS) and healthy controls (HCs).

Clinical and laboratory features	pSS patients	HCs
Number of subjects	44	50
Gender (male/female)	2/42	2/48
Age (years, mean ± SD)	48.43 ± 13.11	47.67 ± 11.93
Duration [month, median (IQ1, IQ3)]	24 (5, 72)	
ESR [mm/h, median (IQ1, IQ3)]	23 (13, 42)	
CRP [mg/L, median (IQ1, IQ3)]	0.88 (0.46, 2.55)	
C3 (g/L, mean ± SD)	1.03 ± 0.20	
C4 [g/L, median (IQ1, IQ3)]	0.16 (0.12, 0.19)	
IgG [g/L, median (IQ1, IQ3)]	18.22 (16.11, 21.51)	
IgA (g/L, mean ± SD)	3.02 ± 1.24	
IgM [g/L, median (IQ1, IQ3)]	1.22 (0.85, 1.64)	
Rheumatoid factor (IU/ml, mean ± SD)	134.57 ± 96.54	
Anti-SSA antibody [median (IQ1, IQ3)]	93 (74.75, 106.25)	
Anti-SSB antibody [median (IQ1, IQ3)]	37 (6.50, 79.50)	
Anti-Ro52 antibody [median (IQ1, IQ3)]	108 (89.25, 117.75)	
ANA titer (range)	1:80-1:1280	
WBC (10^9^/L, mean ± SD)	4.46 ± 1.47	
RBC (10^12^/L, mean ± SD)	4.15 ± 0.46	
HGB (g/L, mean ± SD)	123.79 ± 14.34	
PLT (10^9^/L, mean ± SD)	214 ± 70.97	
Lymphocytes [10^9^/L, median (IQ1, IQ3)]	1.41 (1.14, 1.86)	
Lymphocytes (%, mean ± SD)	35.13 ± 9.76	
Monocytes [10^9^/L, median (IQ1, IQ3)]	0.28 (0.23, 0.36)	
Monocytes (%, mean ± SD)	7.06 ± 1.79	
Neutrophils [10^9^/L, median (IQ1, IQ3)]	2.16 (1.75, 3.07)	
Neutrophils (%, mean ± SD)	54.94 ± 10.33	
ESSDAI score [median (IQ1, IQ3)]	3 (2, 4)	

### Blood sample collection and total RNA isolation

To isolate PBMCs from the fresh blood samples, we used the Ficoll density gradient centrifugation method. Then, the RNA-Quick Purification Kit (EScience Biotech, Tianjin, China) was utilized to extract the total RNA of PBMCs. A NanoDrop2000c spectrophotometer (NanoDrop Technologies, Wilmington, DE, USA) was used to measure the concentration of the total RNA.

### RT-qPCR

Complementary DNA (cDNA) was generated from the reverse transcription of the total RNA using a Bestar™ qPCR RT Kit (DBI Bioscience, Ludwigshafen, Germany). The cDNAs were processed by Bestar^®^ SybrGreen qPCR Master Mix (DBI Bioscience, Ludwigshafen, Germany) and LightCycler^®^ 480 (ABI, Foster City, CA, USA). We used β-Actin as an internal control. The data were analyzed using the 2^–Δ^ ^Δ^ ^Ct^ method. The sequences of amplification primers for METTL3, WTAP, RBM15, ALKBH5, FTO, YTHDF1, YTHDF2, YTHDF3, YTHDC1, YTHDC2, ISG15, USP18, and β-Actin are listed in Table 2. The m6A writers, erasers, and readers used in this study were listed in Supplementary Table 2.

**TABLE 2:** The amplification primers sequences.

Gene name	Sequence (5′–3′)
METTL3	F: AGATGGGGTAGAAAGCCTCCT
	R: TGGTCAGCATAGGTTACAAGAGT
WTAP	F: ACATCCTTGTAATGCGACTAGCAA
	R: ACCATTGTTGATCTCAGTTGGG
RBM15	F: GGATGAGATTTCACCCGAGG
	R: TCTGTGATGACTCCAAAGCGAT
ALKBH5	F: CGCCGTCATCAACGACTACC
	CCCGAATAGGCTTGAACTGGA
FTO	F: GCCCGAACATTACCTGCTG
	R: TGCTCCTTCTAGGGTTTTGCT
YTHDF1	F: TAACGACAACAAACCGGTCACA
	R: ATGGAGGTTGTGTGCTTGTAGG
YTHDF2	F: AGCCCCACTTCCTACCAGATG
	R: TGAGAACTGTTATTTCCCCATGC
YTHDF3	F: ATCAGAGTAACAGCTATCCAC
	R: CCCAGGTTGACTAAATACAC
YTHDC1	F: AAGGAGGGCCAAATCTCCTA
	R: CAGTGTTGTTCCCTTGCTCA
YTHDC2	F: GCAAGAAGAGAAACAACAAACCAC
	R: GCATCACCACCATCATCCAGTA
USP18	F: TTGGGCTCCTGAGGCAAATC
	R: CAACCAGGCCATGAGGGTAG
ISG15	F: CTGAGAGGCAGCGAACTCAT
	R: AGCATCTTCACCGTCAGGTC
β-actin	F: TGACGTGGACATCCGCAAAG
	R: CTGGAAGGTGGACAGCGAGG

### Statistical analysis

If the RT-qPCR data conformed to the normal distribution, Student’s *t*-test was used to analyze it. If the data did not fit the normal distribution, the non-parametric test was used. The correlation analysis was tested through the Pearson method and the Spearman method. We performed binary logistic regression to evaluate whether the m6A elements were the risk factors for pSS. GraphPad Prism (version 8.0) and SPSS (version 26.0) were applied to analyze the data. A two-sided value of *P* < 0.05 was considered a statistically significant difference.

## Results

### Expression of N6-methyladenosine-related genes in peripheral blood monocular cells of patients with primary Sjögren’s syndrome, patients with non-SS sicca, and healthy controls

#### Writers: METTL3, WTAP, and RBM15

**FIGURE 1:**
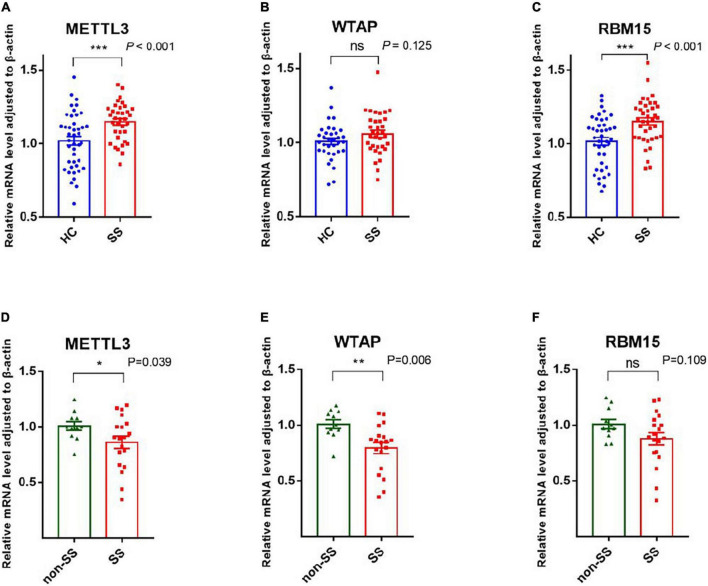
Messenger RNA (mRNA) levels of N6-methyladenosine (m6A) writers in peripheral blood mononuclear cells (PBMCs) from patients with primary Sjögren’s syndrome (pSS), patients with non-SS sicca, and healthy controls (HCs). **(A,D)** The mRNA level of METTL3 in PBMCs from patients with pSS was higher than that of HCs but lower than that of patients with non-SS sicca. **(B,E)** No significant difference was found in the expression of WTAP between patients with pSS and HCs. When compared to patients with non-SS sicca, the mRNA level of WTAP was decreased in patients with pSS. **(C,F)** The mRNA level of RBM15 in PBMCs from patients with pSS was higher than HCs. No significant difference was found in the expression of RBM15 between patients with pSS and patients with non-SS sicca. *p < 0.05, **p < 0.01, and ***p < 0.001.

METTL3, METTL14, and WTAP constitute a multicomponent protein complex that catalyzes m6A RNA methylation, with METTL3 serving as the essential methyltransferase (19). We screened the expression of METTL3, RBM15, and WTAP in PBMCs of patients with pSS, patients with non-SS sicca, and HCs by qRT-PCR. As shown in Figure 1, the mRNA levels of METTL3 and RBM15 in PBMCs of patients with pSS were significantly elevated relative to those of HCs (both *P* < 0.001), although there was no difference in the expression of WTAP (*P* = 0.125). Compared to patients with non-SS sicca, the mRNA levels of METTL3 (*P* = 0.039) and WTAP (*P* = 0.006) in PBMCs of patients with pSS were decreased, whereas there was no difference in the expression of RBM15 (*P* = 0.109) (Figure 1).

#### Erasers: ALKBH5 and FTO

**FIGURE 2:**
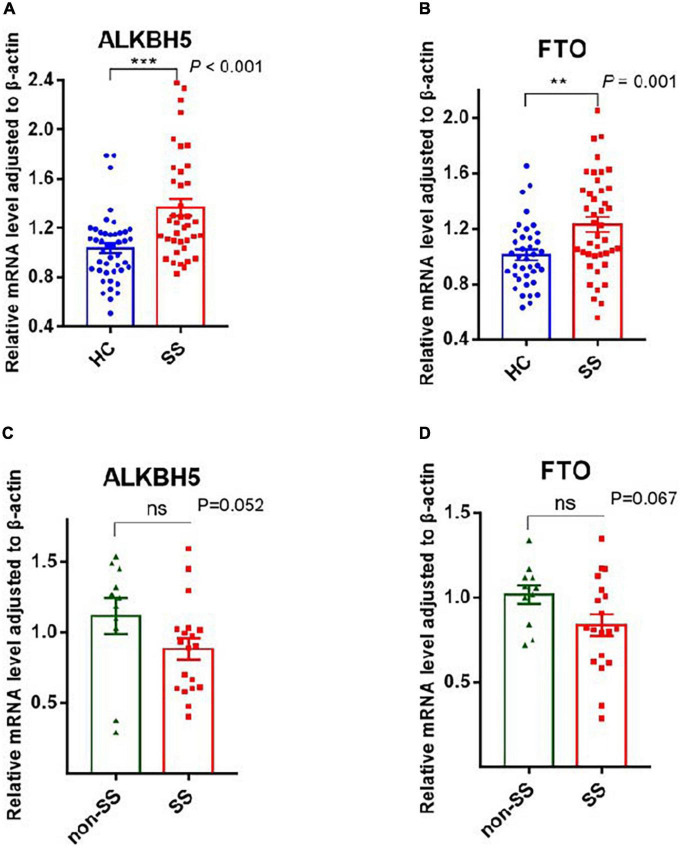
Messenger RNA (mRNA) levels of N6-methyladenosine (m6A) erasers in peripheral blood mononuclear cells (PBMCs) from patients with primary Sjögren’s syndrome (pSS), patients with non-SS sicca, and healthy controls (HCs). **(A,B)** The mRNA levels of ALKBH5 and FTO in PBMCs from patients with pSS were higher than HCs. **(C,D)** No significant difference was found in the expression of ALKBH5 and FTO between patients with pSS and patients with non-SS sicca. **p < 0.01 and ***p < 0.001.

ALKBH5 and FTO, as two demethylases, are responsible for removing the m6A modification from mRNA (20, 21). Our statistics revealed that the mRNA expression of ALKBH5 and FTO was considerably higher in patients with pSS than in HCs, with *P*-values of <0.001 and 0.001, respectively (Figure 2). Compared to patients with non-SS sicca, the expression of ALKBH5 and FTO tended to decrease in PBMCs of patients with pSS but did not reach a statistical significance (Figure 2). Due to the interaction between writers and erasers, the m6A modification is dynamic and reversible. Our results showed that the installation and removal of m6A on mRNAs were increased in patients with pSS, indicating that the m6A process is active.

#### Readers: YTHDF1, YTHDF2, YTHDF3, YTHDC1, and YTHDC2

**FIGURE 3:**
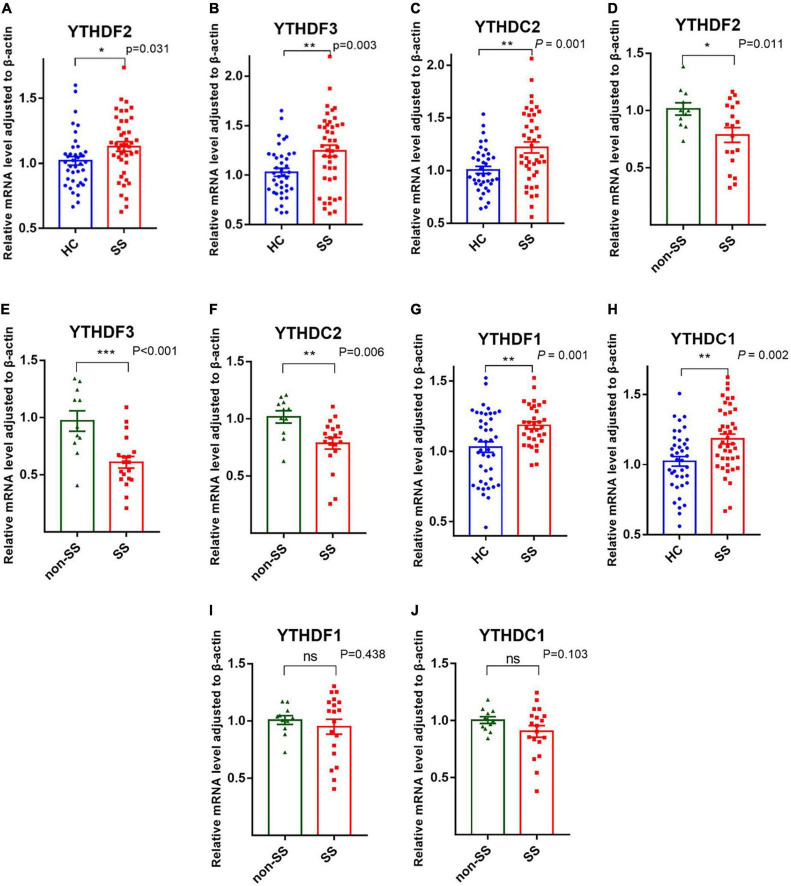
Messenger RNA (mRNA) levels of N6-methyladenosine (m6A) readers in peripheral blood mononuclear cells (PBMCs) from patients with primary Sjögren’s syndrome (pSS), patients with non-SS sicca, and healthy controls (HCs). **(A–F)** The mRNA levels of YTHDF2, YTHDF3, and YTHDC2 in PBMCs from patients with pSS were higher than that of HCs but lower than that of patients with non-SS sicca. **(G–J)** The mRNA levels of YTHDF1 and YTHDC1 in PBMCs from patients with pSS were higher than HCs. No significant difference was found in the expression of YTHDF1 and YTHDC1 between patients with pSS and patients with non-SS sicca. *p < 0.05, **p < 0.01, and ***p < 0.001.

YTHDF1-3, YTHDC1, and YTHDC2, as readers, can precisely identify the m6A modification and are responsible for the specific phenotypic outcomes of m6A-modified mRNAs (22). Our data showed that PBMCs from patients with pSS had elevated mRNA levels of YTHDF1 (*P* = 0.001), YTHDF2 (*P* = 0.031), YTHDF3 (*P* = 0.003), YTHDC1 (*P* = 0.002), and YTHDC2 (*P* = 0.001) compared to HCs (Figure 3). Patients with pSS had lower mRNA levels of YTHDF2 (*P* = 0.011), YTHDF3 (*P* < 0.001), and YTHDC2 (*P* = 0.006) relative to patients with non-SS sicca. No significant difference was found in the expression of YTHDF1 (*P* = 0.438) and YTHDC1 (*P* = 0.103) between patients with pSS and patients with non-SS sicca (Figure 3).

### Correlations between the expression of N6-methyladenosine-related genes and clinical data in patients with primary Sjögren’s syndrome and patients with non-SS sicca

#### Correlations between the expression of N6-methyladenosine-related genes and clinical data in patients with primary Sjögren’s syndrome

**FIGURE 4:**
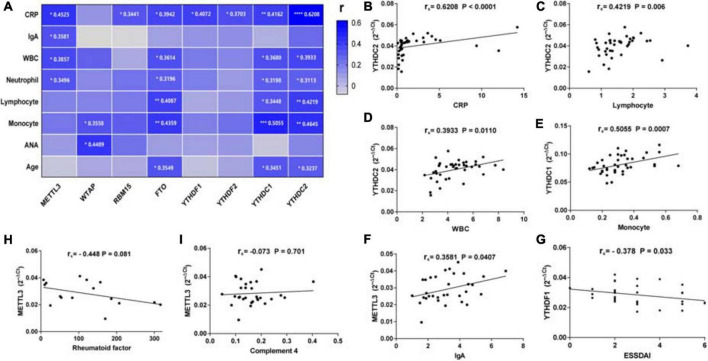
Correlations between the expression of N6-methyladenosine-related genes and clinical data in patients with primary Sjögren’s syndrome (pSS). **(A)** METTL3 was positively correlated with C-reactive protein (CRP), immunoglobulin A (IgA), white blood cells, and neutrophils. WTAP was positively correlated with monocytes and antinuclear antibody (ANA). RBM15, YTHDF1, and YTHDF2 were positively correlated with CRP. FTO, YTHDC1, and YTHDC2 were positively correlated with CRP, white blood cells, neutrophils, lymphocytes, monocytes, and age. **(B–F)** Representative figures of positive correlations between m6A elements and clinical data in pSS. **(G)** The messenger RNA (mRNA) level of YTHDF1 was negatively correlated with the EULAR Sjögren’s syndrome disease activity index (ESSDAI) score. **(H,I)** No significant correlation was found between m6a elements and risk factors for the development of lymphoma in patients with pSS. CRP: C-reactive protein. *p < 0.05, **p < 0.01, ***p < 0.001, and ****p < 0.0001.

To find out whether the mRNA levels of m6A-related genes in PBMCs from patients with pSS could be indicative of disease activity, clinical manifestations and laboratory findings such as ESR, CRP, C3, C4, IgG, IgA, IgM, rheumatoid factor, anti-SSA antibody, anti-SSB antibody, ANA, WBC, RBC, HGB, PLT, lymphocyte, lymphocyte%, monocyte, monocyte%, neutrophil, neutrophil%, disease duration, and ESSDAI score were collected. Then, we performed a correlation analysis between the clinical characteristics patients with pSS and the mRNA levels of m6A-related genes in PBMCs from patients with pSS. As shown in Figure 4, the expression of METTL3 (*r*_*s*_ = 0.4525, *P* = 0.0106), RBM15 (*r*_*s*_ = 0.3441, *P* = 0.0463), FTO (*r*_*s*_ = 0.3942, *P* = 0.0143), YTHDF1 (*r*_*s*_ = 0.4072, *P* = 0.0255), YTHDF2 (*r*_*s*_ = 0.3703, *P* = 0.0203), YTHDC1 (*r*_*s*_ = 0.4162, *P* = 0.0094), and YTHDC2 (*r*_*s*_ = 0.6208, *P* < 0.0001) was all positively correlated with the level of serum CRP level. Interestingly, there was a negative correlation between YTHDF1 mRNA level and ESSDAI score (*r*_*s*_ = −0.378, *P* = 0.033) (Figure 4G). The expression of METTL3 also had positive associations with serum IgA level (*r*_*s*_ = 0.3581, *P* = 0.0407), WBC (*r*_*s*_ = 0.3857, *P* = 0.0266), and neutrophil (*r*_*s*_ = 0.3496, *P* = 0.0416). Moreover, we discovered that the mRNA levels of FTO, YTHDC1, and YTHDC2 were positively correlated with WBC, lymphocyte, monocyte, neutrophil, and age of patients with pSS. In addition, although the mRNA level of WTAP in pSS and HCs did not differ significantly, it was positively associated with monocyte (*r*_*s*_ = 0.3558, *P* = 0.0457) and ANA (*r*_*s*_ = 0.4489, *P* = 0.0166). In addition, the expression of METTL3, WTAP, RBM15, ALKBH5, FTO, YTHDF1, YTHDF2, YTHDF3, YTHDC1, and YTHDC2 in PBMCs of patients with pSS did not correlate with disease duration, ESR, IgG, IgM, C3, C4, anti-SSA antibody, anti-SSB antibody, anti-Ro-52 antibody, RBC, HGB, or PLT (data not shown). Taken together, the expression of METTL3, RBM15, FTO, YTHDF1, YTHDF2, YTHDC1, and YTHDC2 was positively linked with inflammation markers and might reflect the disease activity of pSS.

#### Correlations between the expression of N6-methyladenosine-related genes and clinical data in patients with non-SS sicca

**FIGURE 5:**
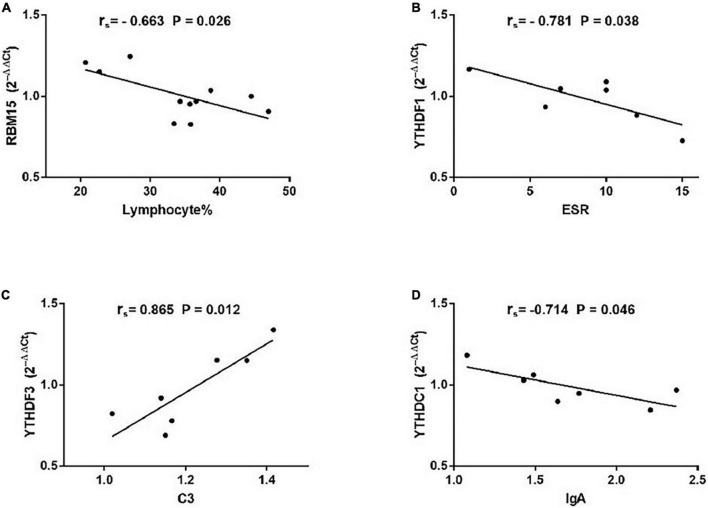
Correlations between the expression of N6-methyladenosine-related genes and clinical data in patients with non-SS sicca. **(A)** The messenger RNA (mRNA) level of RBM15 was negatively correlated with lymphocyte%. **(B)** The mRNA level of YTHDF1 had a negative association with erythrocyte sedimentation rate (ESR). **(C)** The mRNA level of YTHDF3 had a positive association with complement 3 (C3). **(D)** The expression of YTHDC1 was negatively correlated with immunoglobulin A (IgA). ESR, erythrocyte sedimentation rate; C3, complement 3.

As demonstrated in Figure 5, among patients with non-SS sicca, the expression of RBM15 was inversely linked with lymphocyte% (*r*_*s*_ = −0.663, *P* = 0.026). The mRNA level of YTHDF1 was negatively correlated with ESR (*r*_*s*_ = −0.781, *P* = 0.038). The YTHDF3 mRNA level was positively correlated with C3 (*r*_*s*_ = −0.865, *P* = 0.012). The level of YTHDC1 mRNA was negatively associated with IgA (*r*_*s*_ = −0.714, *P* = 0.046). However, the expression of METTL3, WTAP, RBM15, ALKBH5, FTO, YTHDF1, YTHDF2, YTHDF3, YTHDC1, and YTHDC2 in PBMCs of patients with non-SS sicca had no correlation with CRP, C4, IgG, IgM, rheumatoid factor, WBC, RBC, HGB, PLT, lymphocyte, monocyte, monocyte%, neutrophil, or neutrophil%. Together, some m6A elements had associations with ESR and C4 that together reflect the inflammation level in patients with non-SS sicca.

### A risk factor for primary Sjögren’s syndrome: Increased messenger RNA level of ALKBH5 in peripheral blood mononuclear cells

Since the upregulated mRNA levels of METTL3, RBM15, FTO, YTHDF1, YTHDF2, YTHDC1, and YTHDC2 in PBMCs may indicate a higher disease activity of pSS, we applied the binary logistic regression analysis to determine whether m6A-related genes are risk factors for pSS. As exhibited in Table 3, we obtained the equation about the expression of METTL3, RBM15, FTO, YTHDF1, YTHDF2, YTHDC1, and YTHDC2, *Y* = −1.036 × 1 (METTL3) + 4.307 × 2 (RBM15) + 2.630 × 3 (ALKBH5) −0.015 × 4 (FTO) + 2.495 × 5 (YTHDF1) −0.338 × 6 (YTHDF2) + 0.611 × 7 (YTHDF3) + 2.577 × 8 (YTHDC1) + 0.010 × 9 (YTHDC2) −12.819. Based on binary logistic regression, increased expression of ALKBH5 in PBMCs was a risk factor for pSS (*P* = 0.012), whereas other m6A elements were not (*P* > 0.05).

**TABLE 3:** The expression of METTL3, RBM15, ALKBH5, YTHDF1, YTHDF2, YTHDF3 YTHDC1, and YTHDC2 in equation.

	*B*	SE	Wald	*df*	*P*	Exp (*B*)
METTL3	–1.036	3.463	0.089	1	0.765	0.355
RBM15	4.307	2.464	3.055	1	0.080	74.213
ALKBH5	2.630	1.049	6.292	1	0.012	13.876
FTO	–0.015	4.713	0.000	1	0.997	0.985
YTHDF1	2.495	2.771	0.811	1	0.368	12.123
YTHDF2	–0.338	2.395	0.020	1	0.888	0.713
YTHDF3	0.611	1.268	0.232	1	0.630	1.842
YTHDC1	2.577	2.944	0.767	1	0.381	13.164
YTHDC2	0.010	5.265	0.000	1	0.999	1.010
Constant	–12.819	3.727	11.830	1	0.001	0.000

### Correlations between the expression of N6-methyladenosine-related genes and interferon-stimulated genes in primary Sjögren’s syndrome

**FIGURE 6:**
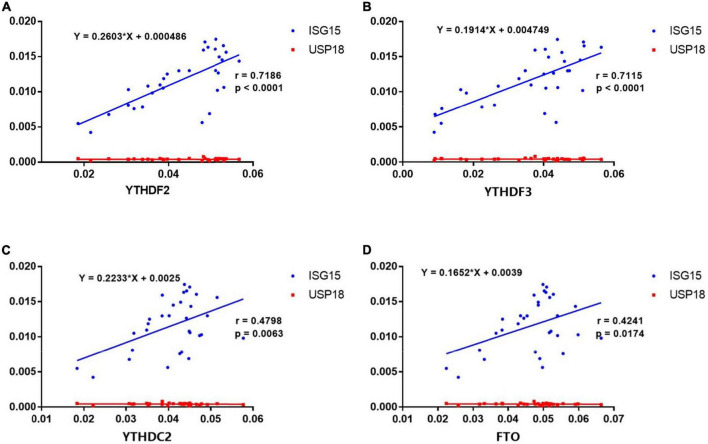
Correlations between the expression of N6-methyladenosine (m6A)-related genes and interferon-stimulated genes (ISGs) in primary Sjögren’s syndrome (pSS). **(A–D)** ISG15 was positively correlated with YTHDF2, YTHDF3, YTHDC2, and FTO in pSS. USP18 was not correlated with YTHDF2, YTHDF3, YTHDC2, and FTO in pSS.

Recent studies have highlighted the critical role of m6A modification in the production of type I interferon (IFN) after viral infection (23–26). To investigate the potential correlations between the m6A modification and ISGs, we detected the mRNA expressions of ISG15 and USP18 in PBMCs from patients with pSS. Then, we performed the correlation analysis between ISGs and METTL3, RBM15, FTO, YTHDF1, YTHDF2, YTHDC1, and YTHDC2. The results demonstrated that the mRNA levels of FTO, YTHDF2, YTHDF3, and YTHDC2 had positive correlations with the expression of ISG15 (Figure 6). Moderate correlations between YTHDF2, YTHDF3, and ISG15 were demonstrated by determinations (*R*^2^) ranging from 0.4888 to 0.8514 and 0.4776 to 0.8513, respectively. The linear equations between FTO, YTHDF2, YTHDF3, and ISG15 are shown in Table 4. These results indicate that the increased expressions of FTO, YTHDF2, YTHDF3, and YTHDC2 may modulate the level of type I IFN and play a potential role in the pathogenesis of pSS. In contrast, no significant correlation was found between m6A and USP18.

**TABLE 4:** The linear equations between FTO, YTHDF2, YTHDF3, and ISG15.

	Equation	*P*-value
FTO	*Y* = 1.089 × *X* + 0.03416	0.0174
YTHDF2	*Y* = 1.984 × *X* + 0.01975	< 0.0001
YTHDF3	*Y* = 2.645 × *X* + 0.005198	< 0.0001
YTHDC2	*Y* = 1.031 × *X* + 0.02891	0.0063

## Discussion

The underlying mechanisms of pSS remain unclear. Multiple factors, including susceptible population, environmental stimuli, and infection, all contribute to the generation of auto reactive lymphocytes and autoimmunity in patients with pSS (26). Increasing evidence suggests that the activation of the type I IFN pathway plays a substantial role in the development of autoimmune diseases (27–34). Accumulated evidence has shown that m6A significantly impacts RNA metabolism and is associated with autoimmune diseases. Interestingly, recent studies have identified m6A as a vital factor in post-transcriptional control of ISG translation during the type I IFN response for antiviral restriction (23, 24, 35–37). Although several studies have described the alterations of m6A levels in RA and SLE, no previous study has investigated the role of m6A in pSS. This is the first study to measure the mRNA levels of METTL3, WTAP, RBM15, FTO, ALKBH5, YTHDF1, YTHDF2, YTHDF3, YTHDC1, and YTHDC2 in PBMCs from patients with pSS and to compare them with those from patients with non-SS sicca and HCs. Moreover, we initially explored the associations between m6A and ISGs. Our data showed that the mRNA levels of METTL3, YTHDF2, YTHDF3, and YTHDC2 in PBMCs from patients with pSS were higher than that of HCs but lower than that of patients with non-SS sicca. The pSS patients’ expression of RBM15, ALKBH5, FTO, YTHDF1, and YTHDC1 was higher than that of HCs but was comparable with that of patients with non-SS sicca. The level of WTAP mRNA in PBMCs from patients with pSS was lower than that of patients with non-SS sicca but was comparable with HCs.

First and foremost, we found that the mRNA level of METTL3 in PBMCs from patients with pSS had positive correlations with serum CRP level, serum IgA, WBC, and neutrophil, which was in accordance with a previous study of RA (12). The level of RBM15 mRNA in PBMCs from patients with non-SS sicca was inversely linked with lymphocyte%. Wang et al. (12) reported that the mRNA level of METTL3 was elevated in PBMCs and monocytes from patients with RA, and it had positive associations with CRP and ESR. Furthermore, Wang et al. (12) demonstrated that METTL3 could reduce LPS-induced inflammation in macrophages *via* NF-κB and play a crucial role in RA. In contrast, Luo et al. (14) reported that the expression of METTL3 was reduced in patients with SLE, especially in those with alopecia. Together, increased expression of METTL3 in pSS may indicate a more severe inflammation response and a higher disease activity in patients with pSS, making it a potential candidate for novel biomarkers for the diagnosis and treatment of pSS.

Second, our data revealed positive correlations between the mRNA level of FTO in PBMCs and laboratory results (CRP, WBC, neutrophil, lymphocyte, and monocyte) in patients with pSS. Interestingly, the mRNA level of FTO was found to be reduced in patients with RA and was positively associated with ESR, CRP, and IgG while negatively correlated with C3 and lymphocyte-to-monocyte ratio (15). In patients with SLE, the expression of ALKBH5 was decreased and was negatively associated with the titer of the anti-dsDNA antibody. ALKBH5 also had correlations with clinical manifestations of patients with SLE, including rash, oral ulceration, and leukopenia (14). Collectively, abnormal expression of m6A erasers has been found in autoimmune diseases, and it was correlated with disease activity. In addition, our data showed that increased mRNA level of ALKBH5 in PBMCs was a risk factor for pSS. Notably, previous studies have reported that downregulated expression of ALKBH5 in PBMCs was a risk factor for RA and SLE (14, 15). These findings indicate that ALKBH5 might play a prominent pathogenic role in the development of pSS.

Third, our study showed that the mRNA levels of m6A readers (YTHDF1 and YTHDF2) were elevated and positively associated with CRP in patients with pSS. Interestingly, the level of YTHDF1 mRNA was negatively correlated with the ESSDAI score in patients with pSS and with ESR in patients with non-SS sicca, indicating that YTHDF1 might act as a negative regulator of inflammation. The YTHDF3 mRNA levels of patients with non-SS sicca were positively correlated with C3. Previous studies have found decreased YTHDF2 expression in RA and SLE (13–15). Luo et al. (14, 15) demonstrated that the expression of YTHDF2 had associations with RBC, lymphocyte%, neutrophil%, neutrophil-to-lymphocyte ratio, and lymphocyte-to-monocyte ratio in patients with RA and had connections with C3, lymphocyte%, neutrophil-to-lymphocyte ratio, and fever in patients with SLE. Moreover, downregulated mRNA level of YTHDF2 in PBMCs was a risk factor for both RA and SLE analyzed by logistic regression (14, 15). In addition, our data showed that readers (YTHDC1 and YTHDC2) were increased in pSS and associated with CRP, WBC, neutrophil, lymphocyte, and monocyte in patients with pSS. Thus, m6A readers are linked to the levels of inflammation indicatives. Patients with pSS are 15 to 20 times more likely to develop B-cell lymphoma than the general population (38, 39). As risk factors for the development of lymphoma in patients with pSS (3), rheumatoid factor, low complement four levels, and a score of >5 on the ESSDAI were unrelated to the expression of m6A elements in PBMCs from patients with pSS.

We have described the alterations of m6A in patients with pSS and demonstrated the correlations between m6A and clinical features, suggesting that m6A elements are risk factors and inflammation indicators in pSS. Nevertheless, the understanding of possible mechanisms of m6A in the pathogenesis of pSS is still limited. Elevated type I IFN activity has been found in PBMCs, saliva, and minor salivary gland biopsies of patients with pSS (26, 29). Hong et al. (25) showed that type I IFN affiliates the production of inflammasomes that have a close connection with pyroptosis in the salivary glands of patients with pSS. Type I IFNs induce hundreds of ISGs. Numerous studies have found that ISGs overexpress in the peripheral blood and salivary glands of patients with pSS (26). The expression of the ISGs in pSS has positive correlations with the titers of anti-SSA and anti-SSB autoantibodies, indicating that ISGs might play a crucial role in the pathogenesis of pSS. In recent years, numerous studies have emphasized the significance of epigenetic regulation of IFN-induced genes in pSS. Through chromatin rearrangements, DNA methylation can influence gene expression (40). Altorok et al. (41) reported that in naïve CD4 + T-cells of pSS, IFN-regulated genes (STAT1, IFI44L, IFITM, and USP18) were hypomethylated. Imgenberg-Kreuz et al. (42) demonstrated that hypomethylation of IFN-regulated genes (MX1, IFI44L, and PARP9) was related to their elevated expression in CD19 + B-cells of pSS. Remarkably, several recent studies have identified the role of m6A in the regulation of ISGs. McFadden et al. (23) uncovered that METTL3, METTL14, and YTHDF1 were capable of upregulating the expression of IFITM1 (belonging to ISGs), thus, promoting the antiviral activity of type I IFN. McFadden et al. (35) revealed that FTO is a negative regulator of STAT3-mediated signaling that induces pro-inflammatory ISGs during the IFN response, addressing an essential role for FTO in suppressing inflammatory genes. Zhang et al. (24) observed elevated ISG levels in YTHDF3−/− mice that were insusceptible to various viral infections. Furthermore, they have revealed that YTHDF3 facilitates the translation of FOXO3 mRNA and negatively regulates IFN-dependent antiviral immunity. Therefore, the mRNA levels of USP18 and ISG15 in PBMCs from patients with pSS were detected in our study. We found that the expression of ISG15 had moderate correlations with the mRNA levels of FTO, YTHDF2, YTHDF3, and YTHDC2, suggesting that the expression of ISG15 might be modulated by the m6A process.

ISG15, strongly induced by type I IFNs, whose secretion was increased in patients with pSS, is an IFN-induced protein and has been identified as a pivotal player in the host antiviral response (25). ISG15 can be induced by multiple factors, including viral and bacterial infections, lipopolysaccharide, retinoic acid, or specific genotoxic stressors (43). ISG15 has been found to influence the functions of several different types of cells. ISG15 induces NK cell proliferation, prompts NK cells to produce IFN-γ, stimulates T-cells to secrete IFN-γ, and promotes macrophages to secrete pro-inflammatory cytokines (44–46). Moreover, ISG15 promotes the maturation of DC and upregulates its expression of E-cadherin, CD15, and CD86 (47). Based on the moderate associations between the ISG15 mRNA level and the mRNA levels of FTO, YTHDF2, YTHDF3, and YTHDC2 in patients with pSS, we hypothesize that ISG15 might be a target of m6A elements in patients with pSS. The ISG15 mRNA might be hypomethylated by m6A eraser FTO and its expression was increased in patients with pSS compared to HCs. Then, the hypomethylated ISG15 mRNA can be recognized by m6A readers YTHDF2, YTHDF3, or YTHDC2, which alters the stability of ISG15 mRNA and increase the expression of ISG15 in patients with pSS. The upregulated ISG15 can activate immune cells and induce an inflammation response, causing immune dysfunction in patients with pSS. Taken together, we hypothesize that m6A-related genes (FTO, YTHDF2, YTHDF3, or YTHDC2) have the potential to stimulate the expression of ISG15 and activate the type I IFN signaling pathway, playing an active role in initiating the autoimmunity in pSS.

Our study also has limitations. The comparison of RNA epigenetic modifications in the affected salivary glands was not shown. Therefore, we would recruit more tissue samples to explore the m6A modifications in our future study.

## Conclusion

The mRNA levels of METTL3, YTHDF2, YTHDF3, and YTHDC2 in PBMCs from patients with pSS were higher than that of HCs but lower than that of patients with non-SS sicca. The expression of RBM15, ALKBH5, FTO, YTHDF1, and YTHDC1 of patients with pSS was higher than that of HCs but was comparable with patients without SS. The level of WTAP mRNA in PBMCs from patients with pSS was lower than that of patients with non-SS sicca but was comparable with HCs. Several m6A elements are correlated with clinical variables and may indicate the disease activity and inflammation status of pSS. In addition, increased ALKBH5 was a risk factor for pSS. The expression of ISG15 was positively correlated with the expression of FTO, YTHDF2, YTHDF3, and YTHDC2 in patients with pSS. We hypothesize that the expression of ISG15 might be modulated by m6A modification and plays an active role in the pathogenesis of pSS.

## Data availability statement

All data generated or analyzed during this study are included in this article/Supplementary material, further inquiries can be directed to the corresponding authors.

## Ethics statement

The studies involving human participants were reviewed and approved by the Ethics Committee of the Peking Union Medical College Hospital. The patients/participants provided their written informed consent to participate in this study.

## Author contributions

JZ and YF supervised the work and revised the manuscript. QX performed the experiments, analyzed the data, and wrote the manuscript. XW designed the study and was responsible for the outline of the article. CD, LZ, LP, WZ, and YZ optimized and proofread the manuscript. All authors read and approved the submitted version.
