# Corrigendum: The potential role of RNA N6-methyladenosine in primary Sjögren's syndrome

**DOI:** 10.3389/fmed.2023.1251795

**Published:** 2023-07-11

**Authors:** Qiufeng Xiao, Xunyao Wu, Chuiwen Deng, Lidan Zhao, Linyi Peng, Jiaxin Zhou, Wen Zhang, Yan Zhao, Yunyun Fei

**Affiliations:** ^1^Department of Rheumatology and Clinical Immunology, Peking Union Medical College Hospital, Chinese Academy of Medical Sciences and Peking Union Medical College, Beijing, China; ^2^National Clinical Research Center for Dermatologic and Immunologic Diseases, Ministry of Science and Technology, Beijing, China; ^3^State Key Laboratory of Complex Severe and Rare Diseases, Peking Union Medical College Hospital, Chinese Academy of Medical Sciences and Peking Union Medical College, Beijing, China; ^4^Key Laboratory of Rheumatology and Clinical Immunology, Ministry of Education, Beijing, China; ^5^Clinical Biobank, Department of Medical Research Center, Peking Union Medical College Hospital, Chinese Academy of Medical Sciences and Peking Union Medical College, Beijing, China

**Keywords:** primary Sjögren's syndrome, N6-methyladenosine, METTL3, ALKBH5, YTHDF readers, ISG15

In the published article, there was an error in affiliation 1. Instead of “Department of Rheumatology and Clinical Immunology, Chinese Academy of Medical Sciences and Peking Union Medical College, Beijing, China”, it should be “Department of Rheumatology and Clinical Immunology, Peking Union Medical College Hospital, Chinese Academy of Medical Sciences and Peking Union Medical College, Beijing, China”.

The authors apologize for this error and state that this does not change the scientific conclusions of the article in any way. The original article has been updated.

